# Loss of *Prune* in Circadian Cells Decreases the Amplitude of the Circadian Locomotor Rhythm in *Drosophila*

**DOI:** 10.3389/fncel.2019.00076

**Published:** 2019-03-01

**Authors:** Wenfeng Chen, Yongbo Xue, Lisa Scarfe, Danfeng Wang, Yong Zhang

**Affiliations:** ^1^Institute of Life Sciences, Fuzhou University, Fuzhou, China; ^2^Department of Biology, University of Nevada, Reno, Reno, NV, United States; ^3^Institute of Applied Ecology, Fujian Agriculture and Forestry University, Fuzhou, China

**Keywords:** *prune*, TFAM, mitochondrial dysfunction, circadian rhythms, *Drosophila*

## Abstract

The circadian system, which has a period of about 24 h, is import for organismal health and fitness. The molecular circadian clock consists of feedback loops involving both transcription and translation, and proper function of the circadian system also requires communication among intracellular organelles. As important hubs for signaling in the cell, mitochondria integrate a variety of signals. Mitochondrial dysfunction and disruption of circadian rhythms are observed in neurodegenerative diseases and during aging. However, how mitochondrial dysfunction influences circadian rhythm is largely unknown. Here, we report that *Drosophila prune (pn)*, which localizes to the mitochondrial matrix, most likely affects the function of certain clock neurons.Deletion of *pn* in flies caused decreased expression of mitochondrial transcription factor TFAM and reductions in levels of mitochondrial DNA, which resulted in mitochondrial dysfunction. Loss of *pn* decreased the amplitude of circadian rhythms.In addition, we showed that depletion of mtDNA by overexpression of a mitochondrially targeted restriction enzyme mitoXhoI also decreased the robustness of circadian rhythms. Our work demonstrates that *pn* is important for mitochondrial function thus involved in the regulation of circadian rhythms.

## Introduction

Circadian clocks drive rhythms in animal physiology and behavior, thus enabling anticipation of daily environmental changes. Cellular processes occur in compartments including the nucleus, endoplasmic reticulum, and mitochondria. These organelles display diurnal rhythms in structural properties, functions, and responses to stress (Chedid and Nair, 1972; Chaix et al., 2016). Diurnal rhythms in intracellular organelles serve critical cellular functions. For instance, the circadian clock can regulate transcription, the DNA damage response, and the general architecture of the nucleus (Gery et al., 2006; Koike et al., 2012; Chaix et al., 2016). Temporal coordination of protein folding, the unfolded protein response, and secretion likely reduces proteotoxicity (Zhu et al., 2014; Chaix et al., 2016). Mitochondria generate most of the cell’s supply of adenosine triphosphate (ATP), used as a source of chemical energy. In addition to supplying cellular energy, mitochondria are also involved in other processes such as cell signaling (Chandel, 2014). The anterograde (nuclear to mitochondrial) and retrograde (mitochondrial to nuclear) signaling pathways mediate communications between nucleus and mitochondria (Butow and Avadhani, 2004; Chandel, 2014; Ng et al., 2014).

Recent studies have suggested that circadian clocks control aspects of mitochondrial function including oxidative metabolism, nutrient utilization, and mitochondrial dynamics (Manella and Asher, 2016; de Goede et al., 2018). However, how mitochondrial function feeds back to the circadian clock mechanism has not been well characterized. Aging processes are associated with declines in mitochondrial function and accumulation of abnormal mitochondria (Sun et al., 2016). Decreases in circadian amplitude and other disruptions of circadian rhythms are also observed in aged animals (Brown et al., 2011; Duffy et al., 2015). Moreover, mitochondrial dysfunction is a characteristic of neurodegenerative diseases such as Parkinson’s disease, Huntington’s disease, and amyotrophic lateral sclerosis (Quintanilla et al., 2008; Shi et al., 2010; Bose and Beal, 2016), and disruption of circadian rhythms is also a common feature and an early symptom of neurodegeneration. This indicates that there are potential connections between mitochondrial dysfunction and circadian rhythms.

The fruit fly, *Drosophila melanogaster*, is a powerful model for studies of biological rhythms. In organisms, behavioral or physiological traits can be entrained by environmental cues (i.e., light and temperature) that cycle during 24 h, and cycling of these traits persists in the absence of environmental cues due to the self-sustaining clock (Allada and Chung, 2010; Dubowy and Sehgal, 2017). Under conditions of 12 h of light and 12 h of dark cycling (LD), flies have bimodal activity patterns that peak around dawn and dusk (Allada and Chung, 2010; Dubowy and Sehgal, 2017). Their locomotor activity gradually increases before the lights-on and lights-off transitions, a phenomenon termed anticipation. In constant dark conditions (DD), the evening activity peak persists and reoccurs with a period of near 24 h (Allada and Chung, 2010; Dubowy and Sehgal, 2017). The small ventral lateral neurons (sLNvs), which express the neuropeptide PDF, are the master pacemaker neurons responsible for generating the locomotor rhythms under DD (Helfrich-Förster, 1998; Renn et al., 1999; Stoleru et al., 2007; Nitabach and Taghert, 2008; Yao and Shafer, 2014). The PDF-negative circadian neurons, such as the dorsal lateral neurons (LNds) and the 5th sLNvs, control the evening peak of activity (Allada and Chung, 2010; Dubowy and Sehgal, 2017). The dorsal neuron 1 (DN1s) can integrate light and also sense temperature to regulate locomotor activity and sleep (Zhang et al., 2010; Guo et al., 2016; Yadlapalli et al., 2018).

Periodogram analysis is usually used for the analysis of periodic data (Klarsfeld et al., 2003; Rosato and Kyriacou, 2006). Rhythmic data can be considered as a wave, and mainly parameters of period and power are analyzed (Klarsfeld et al., 2003; Rosato and Kyriacou, 2006). The period refers to the time interval at which a rhythmic event repeat. The power, or amplitude, of a locomotor rhythm is the difference in activity between the peaks and troughs and reflects the robustness of the endogenous rhythm.

Here, we report that *prune* (*pn*), which encodes a cyclic nucleotide phosphodiesterase that localizes to the mitochondrial matrix, is involved in the regulation of circadian rhythm in *Drosophila*. Loss of *pn* in circadian cells reduced levels of the mitochondrial transcription factor TFAM and decreased the mitochondrial DNA (mtDNA) level resulting in mitochondrial dysfunction and a decrease in the amplitude of the endogenous clock rhythms. In addition, we found that overexpression of a restriction enzyme, mitoXhoI, which cuts mtDNA, in circadian neurons decreases the robustness of circadian rhythm. Our work demonstrates a potential link between mitochondrial dysfunction and circadian rhythm.

## Materials and Methods

### Fly Strains

Fly stocks were raised at 25°C on standard cornmeal-molasses-yeast medium. The mutants of *pn*^1^ (BL174) and *pn*^2^ (BL81) were obtained from the Bloomington *Drosophila* Stock Center. These two lines were outcrossed into the *iso*^31^ wild-type line for five generations to change the genetic background. *iso*^31^ is a wild-type strain widely used for circadian behavior studies (Ryder et al., 2004). The following lines were also obtained from the Bloomington *Drosophila* Stock Center: UAS-*pn*-RNA interference (RNAi; BL65920), UAS-*tfam*-RNAi (BL26744), *act5C*-Gal4 (BL4414), *elav*-Gal4; UAS-*Dicer2* (BL25750), sqh-mitoEYFP (BL7194), *GMR*-Gal4 (BL1104), *repo*-Gal4 (BL7415) and *tub*-Gal80^ts^ (BL7018). UAS-mito*XhoI* and *hs-pn* were kindly provided by Hong Xu (National Heart, Lung and Blood Institute, Bethesda, MD, USA; Zhang et al., 2015). UAS-*tfam* was a gift from Joseph Bateman (King’s College London, London, UK; Cagin et al., 2015). NP1-Gal4; UAS-mitoAT1.03 was provided by Yufeng Yang (Institute of Life Sciences, Fuzhou University, China). *tim*-Gal4 (Kaneko and Hall, 2000), *pdf*-Gal4 (Renn et al., 1999; Park et al., 2000) and *tim-*Gal4*;pdf-*Gal80 (Murad et al., 2007) were used to drive in circadian cells.

### Plasmids Construction and Transgenesis

To generate the *pn*^Δ^ fly line using CRISPR/Cas9 system, a single-stranded oligonucleotide, *Pn*-ssODN, was used: 5′-GCCGGAAATTACATCTGGTAATGGGCAACGAATCGTGTGACTTGGACTCCGCCGTTTCGGCCGTCAGATAAGCTTCATCGGGAGCACGACTATGTACCTATACTGAACATTCCTCGCCGGGACTACCCGTTGAAA-3′. A *HindIII* recognition site and a stop codon were introduced into the DDH domain (a domain of predicated phosphoesterases) of *pn* locus with this oligonucleotide.

For chiRNAs (a vector for guide RNA expression under the control of U6 promoter) construction, the two target site sequences were cloned into the pU6-BbsI-chiRNA plasmid (Addgene, #45946) as previously described (Gratz et al., 2014) using the following oligonucleotides:

- PAM1-sense: 5′-CTTCGTAGACAAAAGCCAAAGTGA-3′
- PAM1-antisense: 5′-AAACTCACTTTGGCTTTTGTCTAC-3′
- PAM2-sense: 5′-CTTCGTGTCTACGCGCAGCGTCAT-3′
- PAM2-antisense: 5′-AAACATGACGCTGCGCGTAGACAC-3′

The Pn-ssODN and pU6-BbsI-chiRNA plasmids were mixed and injected into embryos of the *act5C*-Cas9 (BL#54590, *y* M{w[+mC] = *Act5C*-Cas9.P}ZH-2A w[*]) fly line (Rainbow Transgenic Flies).

### Screen for Integration

After injection, a single F0 fly was collected and crossed with a FM7 balancer fly. Ten to fifteen larval or pupal flies from the F1 generation were pooled for genome extraction. The following primers were used for amplification and sequencing:

- F1: 5′- CGAATCGATACGCGCAACTGTTG-3′
- R1: 5′- TCGTGCTCCCGATGAAGCTTATC-3′

The F1 primer flanks the left homologous arm of Pn-ssODN, and R1 hybridizes to the *HindIII* site and stop codon region. For the F1R1 polymerase chain reaction (PCR)-positive vials, 10–20 F1 flies were crossed with the FM7 balancer fly by single cross. When eggs were present in the food, the genome of a single F1 fly was extracted and amplified using F1 and R1 primers. The F1 primer plus another primer R2 that flanks the right homologous arm were used for amplification and sequencing:

- R2: 5′-TGCTTGAAGGATGGACTGCTGTC-3′

The *pn*^Δ^ fly line and its control line *act5C*-Cas9 were outcrossed into the *iso*^31^ wild-type line for five generations to change the genetic background.

### Immunostaining

Adult flies (3–5 days old) were collected and fixed at room temperature for 1 h in 4% formaldehyde diluted in PBT (PBS with 0.1% Triton X-100). The brains were dissected in PBT at indicated time points and fixed in 4% formaldehyde in PBT for 20 min at room temperature. The brains were rinsed and washed with PBT three times (15 min each) and then were blocked in 10% normal goat serum in PBT for 2 h at room temperature. The brains were incubated with primary antibody, mouse anti-PDF C7 (Developmental Studies Hybridoma Bank), diluted 1:200 or rabbit anti-PERIOD (1:1,000) in PBT overnight at 4°C. After three 15-min washes, brains were incubated with secondary goat anti-mouse Cy5 (Jackson Immuno Research) or goat anti-rabbit Alexa Fluor488 (ThermoFisher, Waltham, MA, USA) antibody at 1:200 dilution overnight at 4°C, followed by extensive washes with PBT. Finally, the brains were mounted in the VectaMount Permanent Mounting Medium. Imaging was performed on a Leica Confocal Microscope SP8 system. Confocal images were obtained at an optical section thickness of 1–2 μm.

### Quantitative RT-PCR

Real-time quantitative reverse transcription PCR (qPCR) was conducted with total RNA extracted from 20 to 30 male fly heads using TRIzol as per the manufacturer’s protocol (Life Technologies, Carlsbad, CA, USA). cDNA from a reverse-transcription reaction or genomic DNA containing mtDNA were used as a starting material. qPCR was performed using SYBR Select Master Mix for CFX (Life Technologies, Carlsbad, CA, USA) on the CFX96 Real-Time System (BIO-RAD, Hercules, CA, USA). The following primers were used:

- Actin5C-f: 5′-CAGAGCAAGCGTGGTATCCT-3′
- Actin5C-r: 5′-CTCATTGTAGAAGGTGTGGTGC-3′
- pn-f: 5′-TTTCTACGATTTTTGGCCCAGG-3′
- pn-r: 5′-TCGTTGCCCATTACCAGATGTA-3′
- tfam-f: 5′-TGCAACAAGTTCCCCGTGAT-3′
- tfam-r: 5′-GCTAGGGGCCTGACTTTGTT-3′
- mito-f: 5′-GCTCATCATATATTTACCGTTGGA-3′
- mito-r: 5′-AAAATTTTAATTCCAGTAGGAACTGC-3′
- Gapdh1-f: 5′-GACGAAATCAAGGCTAAGGTCG-3′
- Gapdh1-r: 5′-AATGGGTGTCGCTGAAGAAGTC-3′

### Locomotor Behavior Assay

Unless noted otherwise, all the flies were raised on cornmeal/agar medium as indicated at 25°C under LD. Male flies were monitored using Trikinetics *Drosophila* Activity Monitors at temperature indicated under LD for 3–4 days, followed by 6–7 days in DD. Activity records were collected in 1-min bins and analyzed using the Fly Activity Analysis suite software developed by M. Boudinot as part of the Brandeis Rhythm Package (Klarsfeld et al., 2003). Circadian periods were determined by periodogram analysis. The periodograms correspond to 5 days of activity in constant darkness, beginning 24 h after the last light-off transition. Amplitude of the rhythm is defined as the height of the main peaks above the line corresponding to the 95% confidence limit (Klarsfeld et al., 2003). Rhythmic flies were defined by periodogram analysis with the following criteria (filter on): amplitude ≥20, width ≥2 h. Amplitude and width are the height and width of the periodogram peak, respectively. A Matlab-based signal-processing toolbox, flyplot, was used to generate figures (Levine et al., 2002).

### Survival Analysis for Paraquat Treatment

The paraquat toxicity assay was performed on 15-day-old male flies and kept on sucrose agar food (5% sucrose and 2% agar and 10 mM paraquat). For the control and *pn* mutant flies, we made three vials at a time for each genotype. Each vial contains 10–20 flies. The flies were transfer from normal food to paraquat containing food at ZT0 and then counted for living flies every 12 h until all death. Two independent repeats were performed and combined to analysis together. Gehan-Breslow-Wilcoxon test was performed to do curve comparison.

### Statistical Analysis

All statistical analyses were conducted using Graph Pad Prism 7. Normally distributed data were analyzed with two-tailed, unpaired Student’s *t*-test.

## Results

### Loss of *pn* Decreases the Robustness of Circadian Rhythm

**Figure 1:**
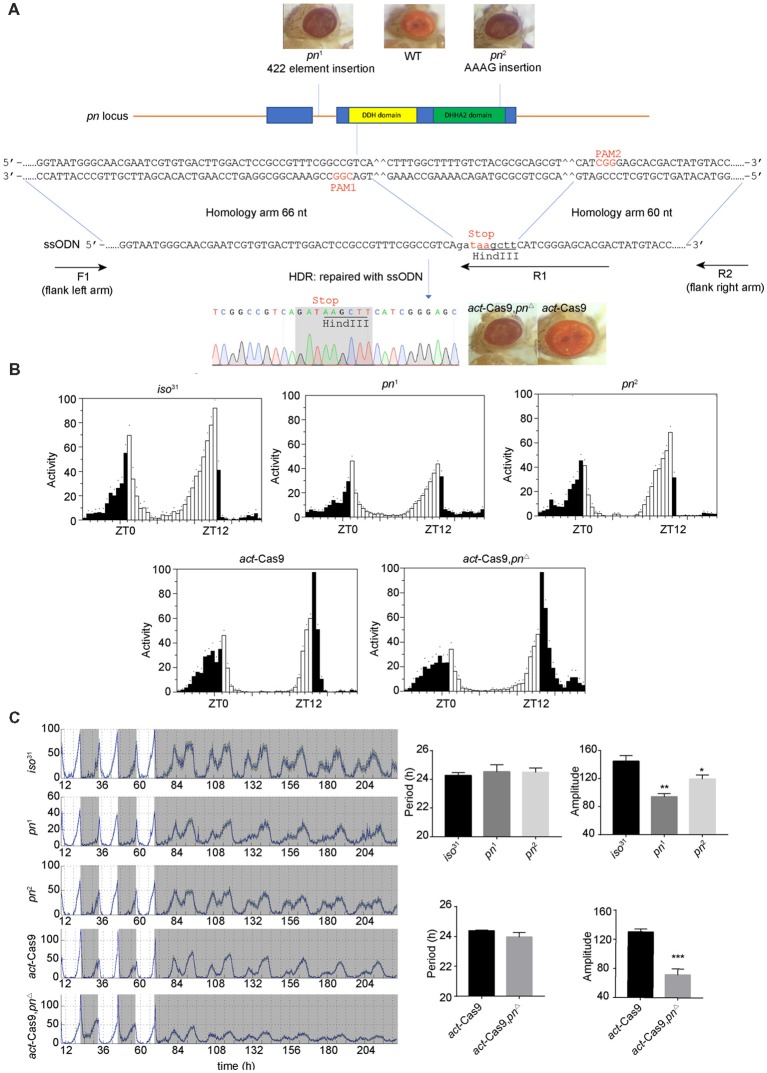
Loss of prune (*pn*) decreases the robustness of circadian rhythm. **(A)** Strategy for generating *pn*^Δ^ mutant. The two target sites located in the DDH domain (a domain of predicated phosphoesterases) are shown. The *pn*-ssODN donor containing two homology arms was designed to introduce a stop codon and delete some bases of *pn* locus. *pn*^1^ is an allele that contains a 422 elements insertion in the first intron of *pn*. *pn*^2^ is an allele that contains an AAAG insertion at the end of DHHA2 domain (a domain often associated with the DHH domain and is diagnostic of DHH subfamily two members). F1, R1, and R2 are the primers used in molecular screening. **(B)** Averaged activity profiles of *pn* mutants during LD. White bars represent day, black bars represent night, Zeitgeber time (ZT) is indicated on the *x* axes. The dots above the bars indicate standard errors of the means (SEM). **(C)** Actograms, period, and amplitude of *pn* mutants and controls. The period and amplitude are shown as means ± SEM (*n* = 32–40). *P < 0.05, **P < 0.01, ***P < 0.001.

*pn* was previously shown as an oscillating gene in fly head (Claridge-Chang et al., 2001). It is required for mtDNA maintenance in mitochondria matrix (Zhang et al., 2015). mtDNA is the small circular DNA located in mitochondria. It encodes 37 proteins that cooperate with the proteins encoded in nuclear DNA to maintain normal mitochondria function (Anderson et al., 1981). To determine the effects of mitochondrial dysfunction on circadian rhythms, we obtained previously described *pn* mutant flies, *pn*^1^ and *pn*^2^ (Frolov et al., 1994; Simmons et al., 1994). To rule out potential effects of genetic background, we also employed CRISIPR/Cas9 to generate a new *pn* mutant line (*pn*^Δ^) by introducing a stop codon in the DDH domain (Figure 1A). As previously reported, both the *pn*^1^ and *pn*^2^ mutants, as well as our newly generated *pn*^Δ^ flies have brownish-purple compound eye phenotype (Figure 1A) that was due to the reduction of drosopterins (Evans and Howells, 1978; Kim et al., 2013). Since there are no *Pn* antibodies available, we used quantitative RT-PCR to determine *pn* abundance in the fly head. We found that *pn* was significantly reduced in both *pn*^1^ and *pn*^Δ^ mutants (Supplementary Figures S1A,B). To minimize the effect of genetic background on behavior rhythms, we crossed the *pn* mutants into *iso*^31^, a wild-type strain widely used for circadian behavior studies (Ryder et al., 2004). We then tested the circadian locomotor behavior rhythms and found that *pn* mutant flies have normal bimodal activity patterns and anticipation under LD (Figures 1B,C). Under DD, the circadian periods and percentages of rhythmic flies were indistinguishable between mutants and control flies. However, the amplitudes of rhythms were reduced in *pn* mutants compared to control, which indicates loss of the robustness of circadian rhythm (Figure 1C). Together, these results indicate that *Pn* is involved in circadian rhythm regulation and loss of *Pn* decreases the robustness of the endogenous clock.

### Adulthood-Specific Overexpression of *pn* Rescues the Decrease in Amplitude of Circadian Rhythms in *pn* Mutants

**Figure 2:**
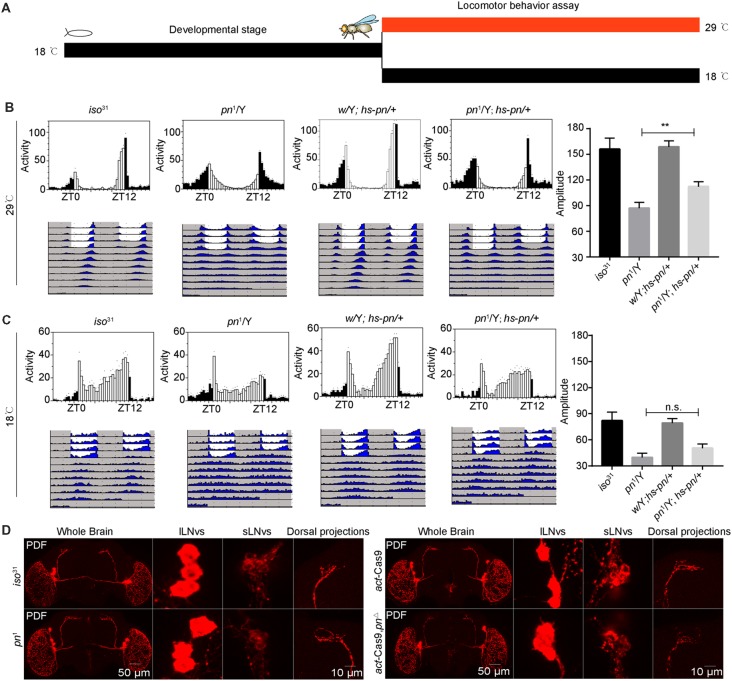
*Pn* functional in adulthood to maintain the amplitude of the circadian rhythm. **(A)** A schematic to show adulthood specific rescue experiments. The flies were reared at 18°C, and then were either transferred to 29°C or kept at the low temperature, and a locomotor activity behavior assay was performed at eclosion. **(B)** Averaged activity profiles and amplitude analysis of flies transferred to 29°C. **(C)** Averaged activity profiles and amplitude analysis of flies maintained at 18°C. White bars represent day, black bars represent night, ZT is indicated on the *x* axes. The dots above the bars indicate SEM. The period and amplitude are shown as means ± SEM (*n* = 30–48). **P < 0.01, n.s. no significance. At least three independent repeats were performed. **(D)** *pn*^1^, *pn*^Δ^ mutant and control brains stained with PDF demonstrate that development of ventral lateral neurons (LNvs) and dorsal projections are normal (*n* = 8–10).

To characterize when *pn* is required for the control of circadian rhythm, we performed an adulthood-specific rescue experiment. We employed a transgenic line expressing *pn* under the control of a temperature-inducible *hsp70* promoter (*hs*-*pn*) and did behavior assay as described in a previous study (Ewer et al., 1988). The flies were reared at 18°C, and then were either transferred to 29°C or kept at the low temperature for locomotor activity behavior assay at eclosion (Figure 2A). At 29°C, *hs-pn* increased the *pn* expression about two fold relative to levels in the control (Supplementary Figure 1C). When the *pn*^1^; *hs*-*pn* flies were raised at a low temperature, transferred to 29°C, and monitored at this higher temperature under DD, the amplitude of locomotor activity rhythms was partially rescued (Figure 2B). In contrast, when flies were raised and tested at the low temperature, the locomotor activity was similar in *pn*^1^; *hs*-*pn* and *pn*^1^ flies (Figure 2C).

Next, we stained the *pn^1^* and *pn*^Δ^ mutant flies with anti-PDF to check the PDF neurons and their projections. We observed that the cell bodies and axonal projections of PDF-expressing circadian pacemaker neurons were indistinguishable to those of controls (Figure 2D). This indicates that abnormal development of PDF neurons is not responsible for the phenotype of the *pn* mutant. Together these results suggest that *pn* is necessary for sustaining robust rhythms in free-running circadian behaviors in the adult.

### Depletion of *pn* in Circadian Tissues Decreases the Robustness of Circadian Rhythm

**Figure 3:**
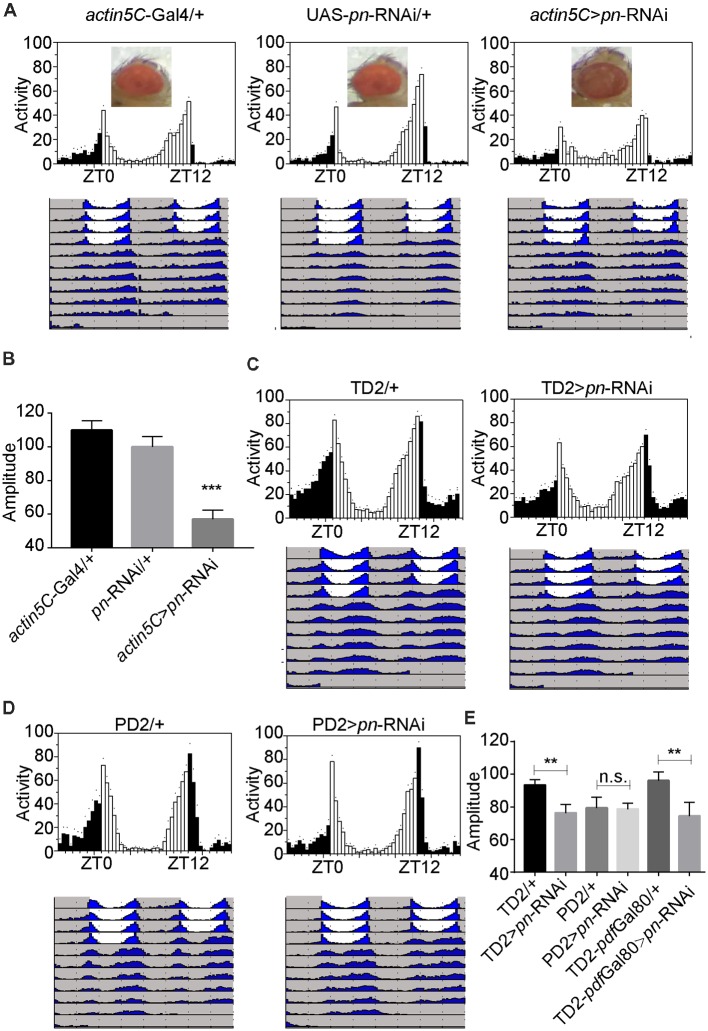
*Pn* expression in circadian tissues affects the robustness of circadian rhythm. **(A)** Averaged activity profiles and actograms of control flies and *actin5C* >*pn*-RNA interference (RNAi) flies. **(B)** Amplitudes of circadian rhythms in control flies and *actin5C* >*pn*-RNAi flies. **(C–E)** Averaged activity profiles, actograms and amplitudes of control flies and flies in which *pn* expression was inhibited by expression of shRNA with different circadian neuron-specific drivers. TD2 indicates *tim*-Gal4, UAS-*Dicer2*. PD2 indicates *pdf*-Gal4, UAS-*Dicer2*. White bars represent day, black bars represent night, ZT is indicated on the *x* axes. The dots above the bars indicate SEM. The period and amplitude are shown as means ± SEM (*n* = 33–50). At least three independent repeats were performed. **P < 0.01, ***P < 0.001, n.s. no significance.

To further identify the cellular requirement of *pn* for circadian rhythms, we used the Gal4-UAS system to drive expression of a mediator of RNAi to downregulate *pn* in specific groups of neurons. When we used *actin5C*-Gal4, a ubiquitous Gal4 driver to express the shRNA targeting *pn*, flies phenocopied the *pn* mutants. These flies had brownish-purple eye color (Figure 3A), which indicates that *pn* expression was suppressed by the shRNA. Furthermore, RT-PCR results showed that *pn* levels were decreased by approximately 60% in the *elav* >*pn*-RNAi flies compared to the GAL4 control flies (Supplementary Figure S1D). As expected, we observed a significant decrease in amplitudes of circadian rhythms when *pn* expression was downregulated (Figures 3A,B).

Downregulation of *pn* in all circadian cells using *tim*-Gal4 resulted in decreased circadian amplitudes, similar to ubiquitous inhibition of expression, which indicates that *pn* is required in circadian cells for the amplitude of rhythms (Figures 3C–E). However, when we depleted *pn* using the *pdf*-Gal4 driver, which is expressed specifically in PDF-positive neurons, no obvious decrease in amplitude was observed (Figures 3C–E). These results suggest that *pn* is required in PDF-negative circadian cells. To confirm this, we combined *tim*-Gal4 with *pdf*-Gal80 to inhibit *pn* expression in PDF-negative circadian neurons. *pdf*-Gal80 is expressed in all PDF-positive LNvs and represses transcription of the UAS in these cells (Murad et al., 2007). We found that *pn* reduction in PDF-negative circadian cells resulted in a decrease in amplitude of circadian rhythms (Figure 3E). Considering that *pn* mutant may impact on visual system and *tim*-Gal4 also expresses in other cells including glia cells, we employed the eye-specific (*GMR*-Gal4) and glia-specific (*repo*-Gal4) Gal4 to knockdown *pn*. No significant reduction of amplitude was observed when *pn* was disrupted in the eye or glia cells (Supplementary Figure S2). These results indicate that Pn is not likely required in fly eyes or glial cells for circadian rhythms.

The decrease of amplitude might be effects of circadian locomotor output or dampening of molecular pacemaker. The molecular pacemaker consists of a negative transcriptional translational feedback loop, in which PERIOD (PER) is one of the main pacemaker proteins (Rosbash, 2009; Hardin and Panda, 2013; Tataroglu and Emery, 2015). Next, we entrained the flies under LD and detected PER abundance in the first day of DD. Our results showed that the PER abundance and oscillation in major circadian neurons of *pn* mutant and control flies are comparable. By contrast, there might be slight change of PER phase in PDF negative circadian neurons, especially in DN1s and LNds in *pn* mutants (Supplementary Figure S3).

Taken together, these results indicate that Pn is required in PDF negative circadian cells and may function in the output of pacemaker to maintain the robustness of endogenous clock.

### Loss of *pn* Causes Mitochondrial Dysfunction

**Figure 4:**
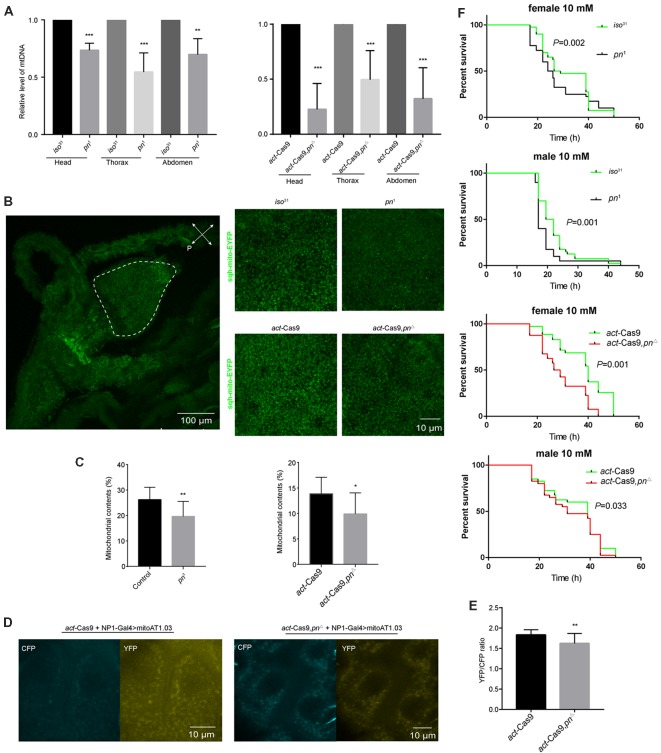
Loss of *pn* causes mitochondrial dysfunction. **(A)** Analysis of mitochondrial DNA (mtDNA) in head, thorax, and abdomen of *pn*^1^, *pn*^Δ^ mutant and control flies. **(B)** Left: image of representative proventriculus cell from the foregut of the 3rd larval of a control fly that expresses mitochondrial label *sqh*-mitoEYFP. Right: representative images of higher magnification proventriculus cells from *pn*^1^, *pn*^Δ^ mutant and control flies labeled with *sqh*-mitoEYFP. **(C)** Comparison of the mitochondrial contents in *pn*^1^, *pn*^Δ^ mutant and control flies. Means ± SD are shown (*n* = 10). *P < 0.05, **P < 0.01, ***P < 0.001. **(D)** Image of representative midgut cells from the 3rd larval of control and *pn*^Δ^ mutant flies labeled with mitoAT1.03 under the driver of NP1-Gal4. **(E)** Comparison of YFP/CFP ratio of mitoAT1.03 in control and *pn*^Δ^ mutant flies. Means ± SD are shown (*n* = 8). **P < 0.01. **(F)** *Pn* mutant and control flies were treated with 10 mM paraquat and were tested for survival curve. Percent survival and times are indicated on the *y* and *x* axes, respectively. Two independent repeats were performed. *P* value are shown on the figures (*n* = 60–80).

*Pn* depletion has been reported to significantly reduce the mtDNA levels in *Drosophila* S2 cells (Zhang et al., 2015). To determine whether the decrease of *pn* causes mtDNA reduction *in vivo*, we analyzed mtDNA levels in three different body segments. We found that there was a significant decline of mtDNA in the head, thorax, and abdomen of *pn* mutant flies relative to control flies (Figure 4A). We next used a ubiquitously expressed EYFP tagged with a mitochondrial-targeting sequence (*sqh*-mitoEYFP; Thomas et al., 2014) to detect the mitochondrial content in *pn* mutant flies. Since we detected significant decreases in mtDNA in the abdomen, we analyzed the foregut cells from the digestive tract, which are large making it easy to detect the mitochondria. In the proventriculus cells from the foregut of the 3rd larval stage, we found that the percentage of cellular area marked for mitochondria was significantly reduced in *pn* mutant flies compared to controls (Figures 4B,C). Moreover, we also employed the ATP sensor (AT1.03) to detect the changes of ATP levels in the midgut cells of *pn* mutant flies (Figures 4D,E). AT1.03 is a fluorescence resonance energy transfer (FRET)-based indicator for ATP that were composed of the ε subunit of the bacterial F0F1-ATP synthase sandwiched by the cyan- and yellow-fluorescent proteins (Imamura et al., 2009). The ratio of YFP/CFP indicates the relative abundance of ATP. As expected, we observed a slightly but significant reduction of ATP levels in the *pn* mutants (Figures 4D,E). Furthermore, *pn* mutant flies were more sensitive than control flies to treatment with paraquat (Figure 4F), a quaternary nitrogen herbicide, which causes oxidative stress and induce mitochondrial dysfunction (Hwang et al., 2012; Kohyama-Koganeya et al., 2017). These data indicate that Pn deficiency causes mitochondrial dysfunction in flies.

### Mitochondrial Dysfunction Decreases the Robustness of Circadian Rhythm

**Figure 5:**
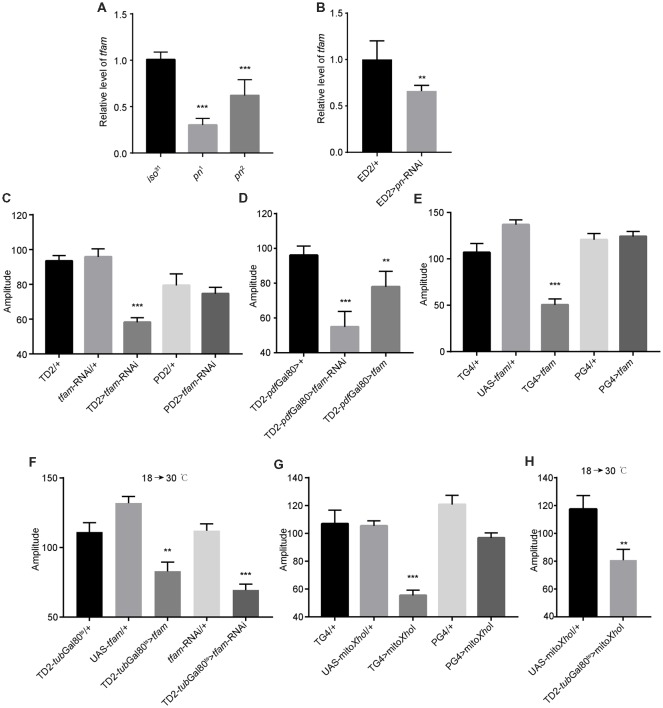
Mitochondrial dysfunction decreases the robustness of circadian rhythm. **(A)** *tfam* mRNA levels in the heads of *pn* mutants and controls. **(B)** *tfam* mRNA levels in the heads of flies in which *pn* is silenced with a pan-neuronal driver and control flies. ED2 indicates *elav*-Gal4; UAS-*Dicer2*. **(C)** Amplitudes of circadian rhythms in flies in which *tfam* has been silenced in the circadian neurons with TD2 or PD2 drivers. **(D)** Amplitudes of circadian rhythms in flies in which *tfam* has been silenced or overexpressed in PDF-negative circadian tissues. **(E)** Amplitudes of circadian rhythms in flies in which *tfam* has been overexpressed in circadian neurons with *tim*-Gal4 or *pdf*-Gal4 drivers. **(F)** Amplitudes of circadian rhythms in flies in which *tfam* was silenced or overexpressed in adulthood stage with *tim*-Gal4 driver. Flies were crossed and reared under 18°C and monitored behavior under 30°C. **(G)** Amplitudes of circadian rhythms in flies in which mitoXhoI has been overexpressed in circadian neurons with *tim*-Gal4 or *pdf*-Gal4 drivers. **(H)** Amplitudes of circadian rhythms in flies in which mitoXhoI was overexpressed in adulthood stage with *tim*-Gal4 driver. Flies were crossed and reared under 18°C and monitored behavior under 30°C. The amplitudes are shown as means ± SEM (*n* = 30–45). At least two independent repeats were performed. **P < 0.01, ***P < 0.001.

The mitochondrial transcription factor TFAM is critical for mtDNA replication (Zhang et al., 2015). Cells depleted of Pn and *pn* mutant flies have lower levels of TFAM protein than relevant control cells or flies, and *tfam* silencing in post-mitotic tissues driven by *GMR*-Gal4 leads to severely deformed eyes in *pn* mutant flies (Zhang et al., 2015). In *pn* mutant fly heads, there were lower levels of *tfam* mRNA than observed in control fly heads (Figure 5A), which is consistent with previous results (Zhang et al., 2015). In addition, when we inhibited expression of *pn* by expression of an shRNA targeting *pn* with a pan-neuronal driver, we also observed decreases in *tfam* (Figure 5B). This indicates that *pn* mutants may cause mitochondrial dysfunction in neurons by influencing TFAM levels.

In order to test this, we downregulated *tfam* in all clock cells by driving *tfam*-targeted shRNA expression with *tim*-Gal4. As expected, we found that depletion of TFAM significantly decreased circadian rhythm amplitude (Figure 5C and Supplementary Figure S1E). Consistent with effects of *pn* downregulation, there was no effect when we restricted *tfam* downregulation to PDF-positive cells using the *pdf-Gal4* driver (Figure 5C). Moreover, we found that *tfam* reduction in PDF-negative circadian cells also resulted in a decrease in amplitude of circadian rhythms (Figure 5D).

It has been shown that excessive TFAM also causes mitochondrial dysfunction by inhibiting mitochondrial gene expression (Falkenberg et al., 2002; Maniura-Weber et al., 2004; Pohjoismäki et al., 2006; Ylikallio et al., 2010). Interestingly, *tfam* overexpression in TIM-positive and PDF-negative neurons also reduced the robustness of clock (Figures 5D,E and Supplementary Figure S1E). In addition, we confirmed that *tfam* dysfunction in adults but not in earlier developmental stages altered locomotor activity (Figure 5F). Thus, TFAM depletion and overexpression both cause mitochondrial dysfunction and reduce the clock robustness.

To further confirm whether other inducers of mitochondrial dysfunction cause the same phenotype as altered TFAM abundance, we manipulated mtDNA levels *in vivo* by expression of a mitochondrially targeted restriction enzyme, mitoXhoI, that cuts the mtDNA once in the *cox I* locus (Cagin et al., 2015). mitoXhoI expression has been demonstrated to cause mitochondrial dysfunction in mice and *Drosophila* (Xu et al., 2008; Ylikallio et al., 2010). We found that mitoXhoI expression driven by *tim*-Gal4 significantly decreased the level of mtDNA in fly heads (Supplementary Figure S1F). Interestingly, mitoXhoI expression in TIM-positive and PDF-negative cells caused reductions in amplitudes of circadian rhythms in the mutants compared to appropriate controls (Figure 5G), a result consistent with the TFAM manipulations. Moreover, we found that mitochondrial dysfunction in the adult but not at earlier developmental stages reduced rhythm robustness (Figure 5H). Thus, mitochondrial dysfunction decreases the robustness of the endogenous circadian rhythm in *Drosophila*.

## Discussion

Circadian systems have three main parts: the core clock, input pathways, and output pathways (Allada and Chung, 2010; Dubowy and Sehgal, 2017). The core clock receives signals from circadian input pathways and then controls the behavior and physiology outputs (Allada and Chung, 2010; Dubowy and Sehgal, 2017). Here, we found that mitochondrial dysfunction in PDF-negative circadian cells affects the amplitude of circadian rhythm. Since PDF-positive cells are the pacemaker neurons, this finding indicates that mitochondrial dysfunction may modulate the output of circadian clock rather than controlling the core circadian system to influence circadian amplitude. Previous work demonstrated that aging is associated with reduced expression of core clock genes in peripheral head clocks in mammals, although similar reductions are not observed in central clock neurons (Duffy et al., 2015). There are two *pn* orthologs PRUNE1 and PRUNE2 in mammals, whether they are involved in the regulation of circadian rhythms need to be studied. It has also been shown that the molecular circadian clock is dampened in peripheral tissues of old flies (Luo et al., 2012; Rakshit et al., 2012; Long and Giebultowicz, 2018). With age, circadian rhythms may lose proper synchronization and become less pronounced. Although we tried different tissue-specific drivers to knockdown *pn*, which cells are involved in the effect of *pn* on the circadian amplitude change need to be determined in the future.

Rhythmic mitochondrial function is known to feed back into the circadian clock mechanism. Changes in mitochondrial function affect the cell at multiple levels, but how the circadian system responds to homeostatic changes is poorly understood. The enzyme SIRT1, an NAD^+^ dependent deacetylase appears to be one link between the clock and aging (Orozco-Solis and Sassone-Corsi, 2014). SIRT1 modulates mitochondrial function by regulating NAD^+^ levels. Interestingly, SIRT1 modulates nuclear-mitochondrial communication and may maintain mitochondrial homeostasis during aging (Gomes et al., 2013; Orozco-Solis and Sassone-Corsi, 2014). In future experiments, it will be interesting to test whether mitochondrial dysfunction is communicated to the circadian clock through SIRT1.

We have confirmed that mitochondrial dysfunction in neurons adversely affects *Drosophila* circadian rhythms. We found that depletion of TFAM specifically in TIM-positive and PDF-negative cells resulted in decreases in circadian amplitude. Moreover, loss of *pn*, which resulted in *tfam* decreases, also caused the same circadian phenotype as *tfam* silencing. Interestingly, both expression of mitoXhoI, which causes degradation of mtDNA, and *tfam* overexpression caused mitochondrial dysfunction and decrease circadian amplitude. This indicates that TFAM mis-expression or direct mtDNA depletion can be used to study the relationship between mitochondria and circadian rhythms.

## Author Contributions

YZ and WC conceived the project, designed the experiments and wrote the manuscript. WC, YX, LS and DW performed the experiments. WC performed the analysis.

## Conflict of Interest Statement

The authors declare that the research was conducted in the absence of any commercial or financial relationships that could be construed as a potential conflict of interest.

## Supplementary Material

FIGURE S1 The results of qPCR analysis. **(A,B)** *pn* mRNA levels in the heads of *pn* mutants and controls. **(C)** *pn* mRNA levels expressed from *hs-pn* heads at indicated temperatures. **(D)** *pn* mRNA levels in the heads of flies in which *pn* is silenced with a pan-neuronal driver and control flies. **(E)** *tfam* mRNA levels in the heads of flies in which *tfam* was silenced or overexpression with the *tim*-Gal4 driver. **(F)** mtDNA levels in the heads of flies in which mitoXhoI was overexpressed. Three independent repeats were performed. **P* < 0.05, ****P* < 0.001. n.s. no significance.

FIGURE S2 *Pn* expression in eyes and glia cells does not affect the robustness of circadian rhythm. (**A**) Averaged activity profiles and actograms of control flies and *GMR*>*pn*-RNAi flies. **(B)** Amplitudes of circadian rhythms in control flies and *GMR*>*pn*-RNAi flies. **(C)** Averaged activity profiles and actograms of control flies and *repo* >*pn*-RNAi flies. **(D)** Amplitudes of circadian rhythms in control flies and *repo* >*pn*-RNAi flies. n.s. no significance. White bars represent day, black bars represent night, Zeitgeber time (ZT) is indicated on the *x* axes. The dots above the bars indicate standard errors of the means (SEM). The amplitudes are shown as means ± SEM (*n* = 40–45). Three independent repeats were performed. n.s. no significance.

FIGURE S3 Oscillation of PERIOD in the circadian neurons of control and *pn*^Δ^ mutant flies. Adult brains were dissected at CT 1, 5, 9, 13, 17, 21 at DD1 and circadian neurons were immunostained with anti-PERIOD. Quantification of average intensity in each clock cell group (*n* = 10) was shown as means ± SEM. Signal was normalized to the value at CT1 which was set as 100%. CT is indicated on the *x* axes. Two independent repeats were performed. **P* < 0.05, ***P* < 0.01, ****P* < 0.001.
