# Supplementary figures and images for: Loss of Prune in Circadian Cells Decreases the Amplitude of the Circadian Locomotor Rhythm in Drosophila
Source: Front Cell Neurosci. 2019 Mar 1;13:76. doi: 10.3389/fncel.2019.00076 (PMC6405476; doi:10.3389/fncel.2019.00076)

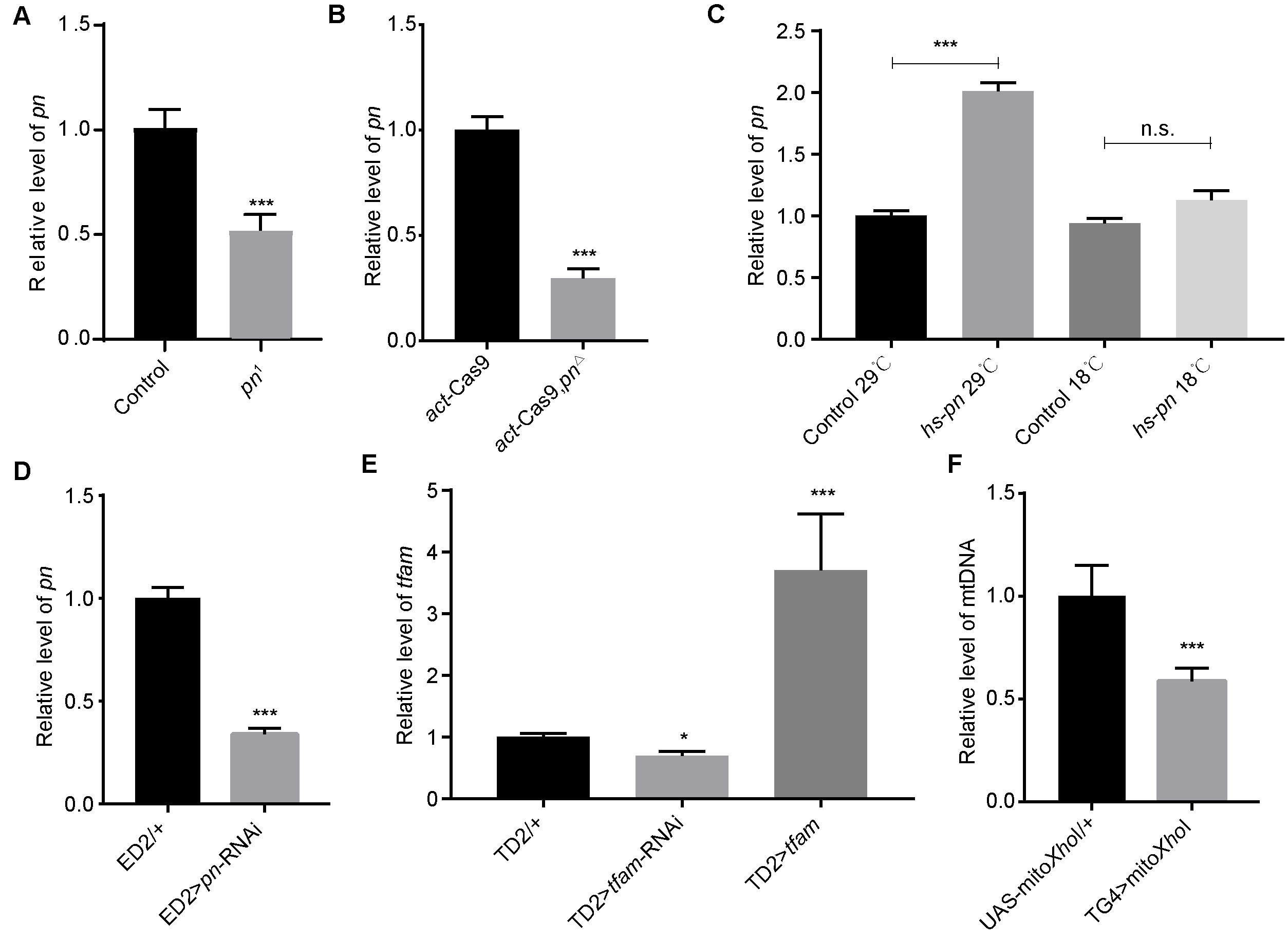

Supplement: FIGURE S1 — The results of qPCR analysis. (A,B) pn mRNA levels in the heads of pn mutants and controls. (C) pn mRNA levels expressed from hs-pn heads at indicated temperatures. (D) pn mRNA levels in the heads of flies in which pn is silenced with a pan-neuronal driver and control flies. (E) tfam mRNA levels in the heads of flies in which tfam was silenced or overexpression with the tim-Gal4 driver. (F) mtDNA levels in the heads of flies in which mitoXhoI was overexpressed. Three independent repeats were performed. *P < 0.05, ***P < 0.001. n.s. no significance. [file Image_1.TIF]

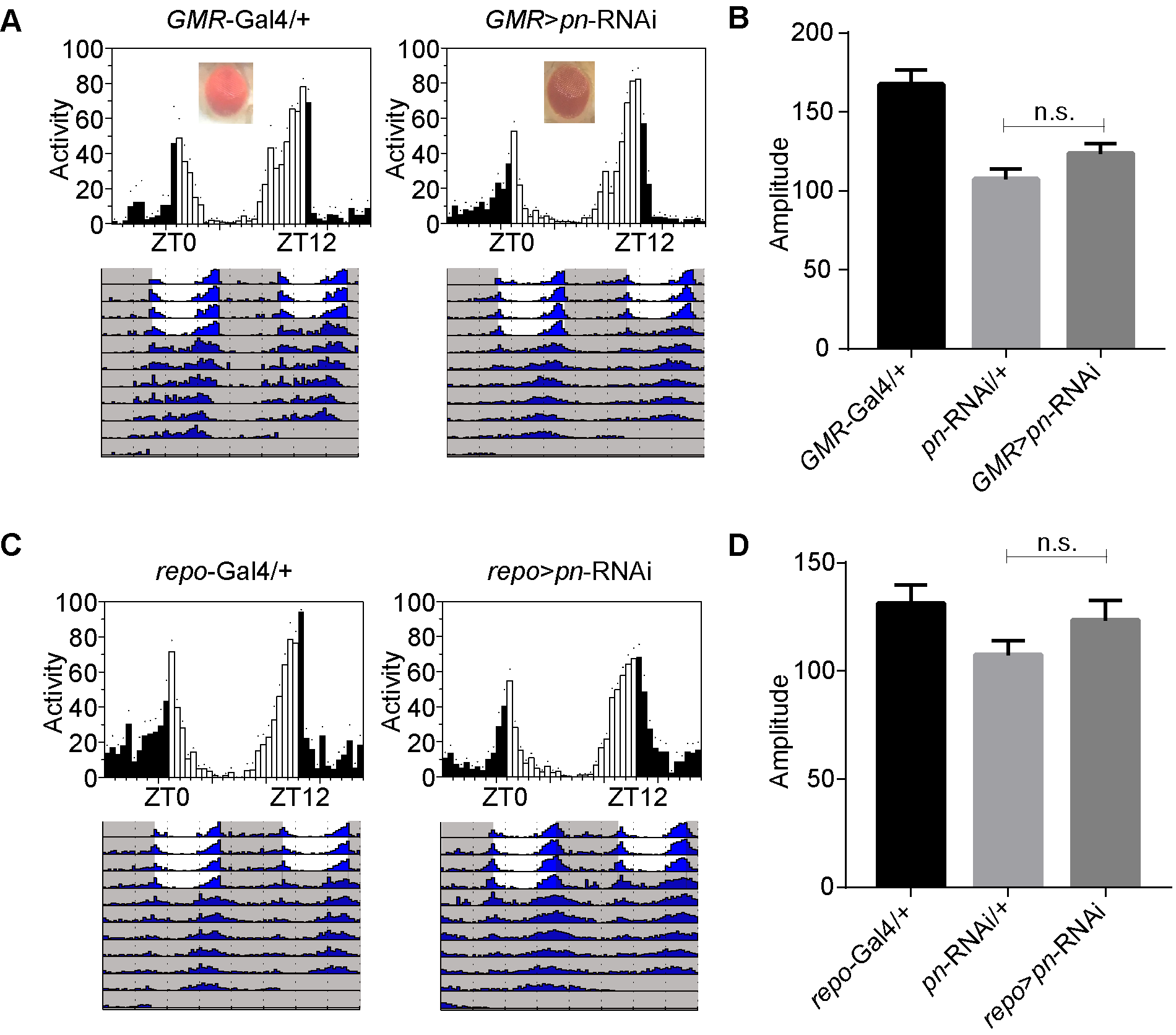

Supplement: FIGURE S2 — Pn expression in eyes and glia cells does not affect the robustness of circadian rhythm. (A) Averaged activity profiles and actograms of control flies and GMR>pn-RNAi flies. (B) Amplitudes of circadian rhythms in control flies and GMR>pn-RNAi flies. (C) Averaged activity profiles and actograms of control flies and repo >pn-RNAi flies. (D) Amplitudes of circadian rhythms in control flies and repo >pn-RNAi flies. n.s. no significance. White bars represent day, black bars represent night, Zeitgeber time (ZT) is indicated on the x axes. The dots above the bars indicate standard errors of the means (SEM). The amplitudes are shown as means ± SEM (n = 40–45). Three independent repeats were performed. n.s. no significance. [file Image_2.TIF]

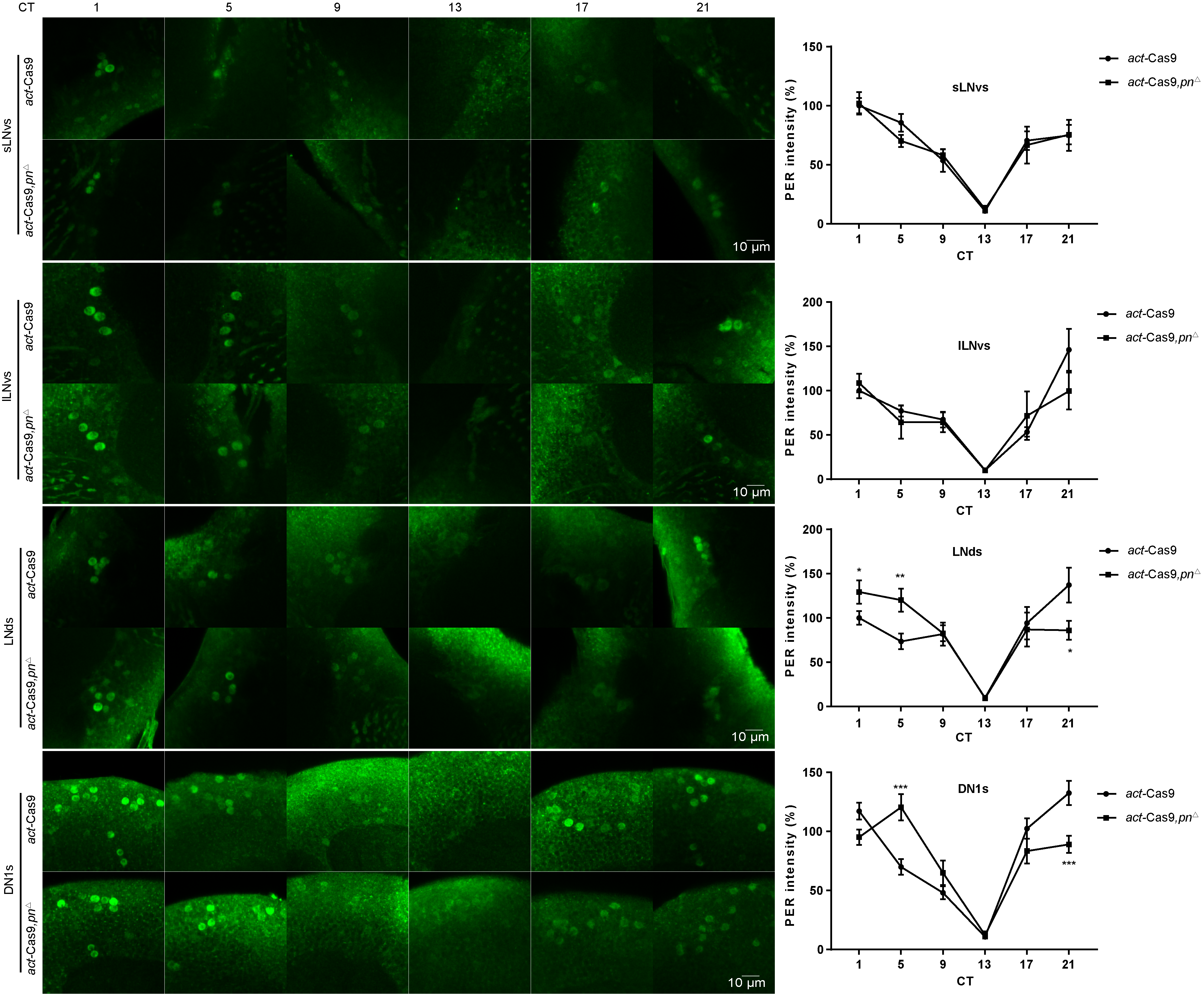

Supplement: FIGURE S3 — Oscillation of PERIOD in the circadian neurons of control and pnΔ mutant flies. Adult brains were dissected at CT 1, 5, 9, 13, 17, 21 at DD1 and circadian neurons were immunostained with anti-PERIOD. Quantification of average intensity in each clock cell group (n = 10) was shown as means ± SEM. Signal was normalized to the value at CT1 which was set as 100%. CT is indicated on the x axes. Two independent repeats were performed. *P < 0.05, **P < 0.01, ***P < 0.001. [file Image_3.TIF]
